# Fruit Juices as a Valuable Ingredient in Isotonic Drinks

**DOI:** 10.3390/foods15172988

**Published:** 2026-08-25

**Authors:** Aleksandra Wilczyńska, Agnieszka Rybowska, Joanna Newerli-Guz, Agnieszka Palka

**Affiliations:** Department of Quality Management, Faculty of Management and Quality Sc, Gdynia Maritime University, 81-225 Gdynia, Poland; a.rybowska@wznj.umg.edu.pl (A.R.); j.newerli-guz@wznj.umg.edu.pl (J.N.-G.); a.palka@wznj.umg.edu.pl (A.P.)

**Keywords:** isotonic drinks, fruit juices, bioactive substances, antioxidant activity, sensory attributes

## Abstract

The growing interest in isotonic drinks translates into the need to develop formulations based on natural raw materials, which limit the use of synthetic additives while simultaneously increasing the bioactive value and sensory appeal of the products. The aim of this study was to develop innovative formulations for isotonic drinks based on natural fruit juices and to evaluate their physicochemical properties and sensory acceptance. Formulations containing 10–15% (*v*/*v*) fruit juice, with the addition of salt (0.13 g/100 mL) and organic sugar, were prepared, taking care to maintain osmolality within the range of 270–330 mOsm/kg. The 10 most appealing variants were selected and subjected to sensory analysis using the QDA; physicochemical analyses, including the determination of total polyphenol content (TP) and the DPPH free radical scavenging capacity; and the measurement of color parameters in the CIE Lab system. All the drinks analyzed received positive ratings in the sensory test (average acceptability scores ranging from 6.9 to 8.1). Physicochemical analyses revealed that the drinks had significant antioxidant activity: polyphenol content ranged from 46 mg to nearly 170 mg GAE/100 mL, whilst DPPH free radical scavenging capacity ranged from 45% to nearly 90%. We found that the use of fruit juices makes it possible to create isotonic drinks which not only effectively rehydrate, but also provide valuable antioxidant compounds.

## 1. Introduction

Isotonic drinks are beverages specifically formulated to enhance athletic performance, support hydration processes, and facilitate the rapid recovery of the body after physical exertion [1,2,3]. This is possible due to their osmolality being close to that of blood plasma (~275–295 mOsm/kg), which aids the quick absorption of fluids and electrolytes and prevents net movement of water across cell membranes, thus maintaining cell shape and function [3,4,5]. The composition of isotonic beverages includes water, carbohydrates, electrolytes, acidity regulators, flavoring and aromatic substances, and colorants. Their proper balance determines the sensory appeal and effectiveness of these beverages [2].

Water, the main ingredient of the beverage, is essential for the proper functioning of the body, including digestion and the regulation of water–electrolyte balance. It plays a particularly important role for physically active individuals, as it supports thermoregulation, the musculoskeletal system, and the transport of oxygen and nutrients. Since the human body cannot store water in large quantities, regular intake is crucial to prevent dehydration [6,7,8,9].

Carbohydrates in isotonic drinks are the primary source of energy. Glucose, sucrose, and maltodextrins are most commonly used, and their optimal concentration (6–8 g/100 mL) allows for the maintenance of isotonicity, enhancement of endurance performance, and good gastric tolerance [10,11,12]. Mixtures of glucose and fructose can increase the efficiency of intestinal transport and reduce the risk of gastrointestinal disturbances [13]. Moreover, their appropriate concentration is often optimal for efficient absorption and ergogenic effects. In contrast, beverages with added fruit juices naturally contribute simple sugars to the product. This necessitates monitoring the osmolality of these products to avoid shifting the product toward a hypertonic beverage [14].

Electrolytes, particularly sodium (300–400 mg/L), play a crucial role in maintaining water–electrolyte balance and preventing hyponatremia [2,15]. The presence of sodium at a concentration of 20–50 mmol/L improves glucose absorption and increases the rate of water absorption. Potassium, magnesium, and calcium have also been studied in isotonic drinks, but their impact remains relatively minor [2,16,17]. The first one supports nerve conduction and muscle function, while the other two are involved in muscle contraction processes and energy metabolism regulation. Although their content in isotonic drinks is lower than that of sodium, they are an important element in maintaining homeostasis during intense physical exertion [2].

Some isotonic drinks are enriched with B vitamins, vitamin C, and bioactive compounds that support metabolic processes or have antioxidant effects. However, sometimes their presence is more of a marketing element than a real physiological necessity. In contrast to energy drinks, isotonic drinks do not contain caffeine [18].

Isotonic formulations are increasingly enhanced with natural plant constituents (polyphenols, anthocyanins, or herbal extracts), reflecting the trend toward natural and functional foods [19]. Currently, there is a growing interest in employing fruit juices as useful ingredients in isotonic beverages. This approach aligns with the “clean label” trend, which promotes replacing synthetic additives with natural raw materials rich in polyphenols, vitamins, anthocyanins, and flavors [20]. Their high content in these raw materials increases the product’s functional value. These compounds exhibit strong antioxidant properties, contributing to the neutralization of free radicals generated during intense physical exertion. The high-dose antioxidant supplementation may attenuate exercise-induced physiological adaptations. Exogenous antioxidants prevent some physiological functions of free radicals that are needed for cell signaling, causing higher dosages of antioxidants to hamper or prevent performance-enhancing and health-promoting training adaptation such as mitochondrial biogenesis, skeletal and cardiac muscle hypertrophy, and improved insulin sensitivity [21]. However, naturally occurring antioxidants supplied by fruits do not appear to produce the same negative effects reported for high-dose antioxidant supplements. The addition of fruit juices may also to improve the sensory qualities of isotonic drinks [6,22,23]. The results of the research by Świtalski and Rybowska [6] confirm the importance of sensory attributes in the acceptance of isotonic drinks. The surveyed consumers indicated that natural fruit flavor and appropriately balanced sweetness are among the key determinants of product choice. Respondents also showed a good attitude toward beverages containing natural extracts, as long as they did not have a negative impact on the product’s taste or clarity.

Grape, apple, and beet juices, which may be ingredients added to isotonic drinks, are natural sources of simple sugars, vitamin C, and phenolic compounds [23,24]. Also, plant extracts such as green tea, yerba mate, hibiscus, mint, and ginger have antioxidant, anti-inflammatory, and adaptogenic properties [22,25,26,27,28,29]. On the other hand, extracts from ginger and turmeric are known for their anti-inflammatory and digestive-supporting potential [30,31]. The addition of hibiscus can have a beneficial effect on blood pressure, and peppermint acts as an antispasmodic and refreshing agent [32,33]. Some extracts, such as yerba mate, exhibit stimulating effects due to their natural caffeine content [1]. Coconut water is an additional natural source of electrolytes, particularly potassium, whereas sugarcane juice contains carbs and trace elements [34].

The components of fruit juices are mainly plant secondary metabolites, which can be divided into polyphenols, polysaccharides, terpenes, alkaloids, proteins, and lipids [35].

The use of fruit juices in isotonic beverages poses a technological challenge: it requires the control of technological parameters such as color stability, the susceptibility of bioactive compounds to oxidation, and the impact of natural sugars on the product’s osmolality.

Fruit juices provide natural simple sugars, vitamins (especially vitamin C), phenolic compounds, and coloring pigments. Their usage in isotonic beverages enhances the sensory value of the product by

- Intensifying aroma and natural sweetness;
- Improving appearance by changing the color of the beverages;
- Increasing acidity and imparting a fresh flavor profile [36].

The combination of plant extracts and fruit juices can lead to synergistic effects, including

- Increased antioxidant stability (for example, vitamin C stabilizes anthocyanins);
- Improvement of the flavor profile and sensory balance;
- Natural coloring of the product;
- Enhanced bioavailability of polyphenols [22,27,37,38].

The literature emphasizes the necessity of balancing the flavor profile of natural isotonic drinks, as intense fruit juices can dominate the fruity note [39].

The market for functional beverages is rapidly growing, and isotonic drinks supplemented with fruit juices, which combine functional benefits with a natural product image, are becoming increasingly popular. They are appreciated not only by professional athletes but also by people engaging in recreational physical activity, those working physically, and consumers seeking an alternative to sweetened carbonated beverages [6]. Consumers are becoming more conscious of not only the health benefits of products, but also their origin, quality certifications, ethical issues, and environmental considerations [27].

From the producers’ perspective, this means the necessity of designing isotonic drinks based on fruit juices, which will serve as “functional–emotional” products, addressing both the physiological and aesthetic needs of consumers. Therefore, the aim of our study was to design and conduct a preliminary evaluation of isotonic drinks based on fruit juices, with particular emphasis on their physicochemical properties and sensory acceptability.

## 2. Materials and Methods

### 2.1. Isotonic Drinks Preparation

Isotonic drink formulations were prepared using different types of 100% fruit juices made from fruit sourced from local suppliers, salt (0.13 g/100 mL) and organic sugar. The quantities of sugar and fruit juices were selected so that the osmolality of the finished isotonic drink was 270–330 mOsmol/kg. Most of the drink formulations were made with between 10 and 15% (*v*/*v*) fruit juice and between 2.5 and 4 g of sugar. All the ingredients were mixed with purified water and pasteurized at 85 °C for 15 min. Each drink was then subjected to an initial organoleptic assessment carried out by a three-person team of experts.

The test samples consisted of 10 isotonic drinks which were selected from among more than 30 pre-prepared isotonic drinks following a preliminary organoleptic assessment, which was designed to identify the most desirable drinks from an organoleptic point of view. Table 1 presents the characteristics of the drinks tested.

These drinks were subjected to both sensory analysis and testing for parameters indicating their biological value: antioxidant activity and total polyphenol content.

### 2.2. Methods

#### 2.2.1. Sensorial Analysis

The criteria adopted for the sensory evaluation were desirability and acceptance of selected indicators of overall quality. The evaluation was carried out under appropriately controlled conditions in a sensory analysis laboratory. The assessment was carried out by a team of 10 selected assessors, who had previously been tested and trained for the purposes of sensory analysis in accordance with the requirements of the standard PN-EN ISO 8586:2014-03 [40]. The study utilized the QDA (Quantitative Descriptive Analysis) palatability profiling method in accordance with ISO 13299:2010 [41]. The samples of isotonic drinks prepared for evaluation were placed in odorless, transparent containers. All were coded with a three-digit number to prevent their identification. Each assessor received a set of 10 samples, each containing 30 mL of the drink. The study assessed the degree of acceptance of 10 quality attributes of the drinks (color, aroma, sweet taste, sour taste, salty taste, bitter taste, fruity taste, clarity, astringency and palatability). In addition, the overall acceptability of the drinks was assessed. An 11-point structured (graphical–numerical) scale was used in the study, with the endpoints “definitely not acceptable” and “definitely acceptable”. The results of the study are presented as the mean (M) together with the standard deviation (SD). Furthermore, the median (Me) and mode (Mo) were calculated.

#### 2.2.2. Color Parameters

Color parameters (L*, a*, b*) were established in the CIE system using a Minolta CR-400 Chroma-meter (Konica-Minolta, Tokyo, Japan).

#### 2.2.3. Osmolality

Measurement of osmolality was performed using a vapor pressure osmometer (Vapro^®^5600, EliTech, San Jose, CA, USA) through direct reading.

#### 2.2.4. Antiradical Activity (AA DPPH)

The scavenging activity against the 1,1-diphenyl-2-picrylhydrazyl hydrate (DPPH) radical of honey was estimated according to the procedure described by Turkmen et al. [42] with some modifications. A 0.75 mL sample of the drinks was mixed with 2.25 mL of 0.1 mM methanol solution of DPPH 1,1–diphenyl-2-picrylhydrazyl (Fluka, Hamburg, Germany). The control test was made with distilled water in place of the drink solution. The reaction mixtures were vortex-mixed well and left in room temperature in the dark for incubation for 60 min. Absorbance was measured at λ = 517 nm against methanol (Fluka, Hamburg, Germany), using a UV-VIS spectrophotometer (Unicam UV2-100, ATi Unicam, Cambridge, UK). The antiradical activity of drinks was expressed as a percentage of inhibition of DPPH radicals and calculated from the equation

(1) AA DPPH [%] = (ABS contr − ABS sample)/ABS contr·100%

#### 2.2.5. Total Phenolic Content (TP)

To determine the total phenolic content of isotonic drinks, the method of Meda et al. [43] was employed. A 0.5 mL sample of the drinks was mixed with 2.5 mL 0.2 N solution of Folin–Cioclateau reagent (Fluka, Hamburg, Germany), and 2 mL of sodium carbonate solution (75 g/L) (POCh, Gliwice, Poland) was added. After incubation in the dark at room temperature for 2 h, the absorbance of the reaction mixture was measured at λ = 760 nm using a UV-VIS spectrophotometer (Unicam UV2-100). The standard curve was produced for gallic acid (Fluka, Hamburg, Germany) within the concentration range from 0 to 200 mg/L. The total phenolic content was expressed as gallic acid equivalents in mg/100 mL of drink sample (mg GAE/100 mL).

### 2.3. Statistical Analysis

The results of physicochemical analysis were analyzed using analysis of variance (ANOVA) and Tukey’s tests in Statistica 13.3 (StatSoft Inc., Tulsa, OK, USA) with *p* < 0.05 and a 95% level of confidence. Also, in order to determine the relationship between the particular parameters, Pearson correlation coefficients (r) were calculated.

## 3. Results

### 3.1. Results of Sensory Analysis

The results of the sensory evaluation are summarized in Table 2. All the fruit juice-based isotonic drinks analyzed were rated positively and met the sensory minimum standards set for innovative products. All samples received high acceptance ratings (M = 6.9–8.1). The isotonic drink based on Japanese quince and elderberry juice (661) received the highest rating (acceptance M = 8.1). This drink also received the highest rating for color and high ratings for taste and other sensory attributes. The isotonic drink based on apple and chokeberry juice (sample 100) received the lowest rating (acceptance M = 6.9). The individual quality attributes of the developed drinks were rated highly (Mo (6–10)). The low standard deviations indicate a consensus among the panel of assessors.

The individual isotonic drinks assessed were characterized by features that distinguished them from the other products:

1. Color: The highest score was given to the color of the isotonic drink made from quince and elderberry juice (sample 661) (M = 9.4), whilst the lowest score was given to the color of the isotonic drink made from raspberry and elderberry juice (sample 443) (M = 6.9), but this rating is still high and the sample is acceptable.
2. Aroma: The aroma of the isotonic drink based on pear and quince juice (sample 336) received the highest rating (M = 8.3), suggesting that it has a well-balanced and pleasant aroma. The aroma of the raspberry juice-based drink (sample 114) (M = 6.8) received the lowest rating.
3. Sweetness: The highest rating was given to the isotonic drink sample based on raspberry and elderberry juice (sample 443) (M = 7.6), which means that, according to the assessors, its sweetness was the most appropriate. The lowest rating for this attribute was given to the isotonic drink based on pear and quince juice (sample 336) (M = 7.0).
4. Sourness: The sourness of the isotonic drink based on cranberry juice (sample 226) (M = 7.9) was rated highest. This may serve as a benchmark for a well-balanced level of acidity in this type of drink. The lowest rating for this attribute was given to the drink sample based on bird cherry and apple juice (sample 889) (M = 7.0).

### 3.2. Results of Physicochemical Analysis

Table 3 shows the average values of the individual physicochemical parameters. The total polyphenol content (TP) in the isotonic drinks studied ranged from just over 46 mg GAE/100 mL in the JŻ drink (apple and cranberry) to 172 mg GAE/100 mL in the JA drink (apple and chokeberry). All the drinks tested exhibited a high free radical scavenging capacity, ranging from 45 to almost 90%, with the JA drink (apple and chokeberry) showing the strongest scavenging activity against DPPH free radicals and the apple and cranberry juice-based drink (JŻ) showing the weakest. An analysis of variance showed that the drinks studied did not differ significantly in their free radical scavenging capacity; however, the total polyphenol content did depend significantly on the type of juice or juices used.

The type of fruit juice used also had a significant impact on the color of the isotonic drinks produced. The drink made from elderberry and Japanese quince juices (5 PCzB) had the lightest color (the highest L* value) and also had the highest values for both the a* and b* parameters, which means that the color of this drink has a high saturation of redness and yellowness. The drink made from cranberry juice (20 Ż), on the other hand, was the darkest in color. Correlation analysis revealed a statistically significant negative dependence between the L* value and the ability to scavenge free radicals DPPH (r = −0.46) and the total phenolic content (TP) (r = −0.58). There was also a statistically significant but weak negative relationship between color redness (a* value) and the total phenolic content (TP) (r = −0.38). We also noticed a significant positive correlation (r = 0.66) between antiradical activity (AA DPPH) and total polyphenol content (TP). All correlations were significant at *p* < 0.05. The correlations observed suggest that the darker and less red a given drink was, the greater its antioxidant potential.

## 4. Discussion

The primary objective of an isotonic beverage is to facilitate rapid rehydration, which is strictly dependent on its osmolality. In our study, the osmolality was carefully adjusted to the range of 270–330 mOsm/kg. This aligns perfectly with the findings of Kayshar et al. [44] regarding Ajwa date-based beverages (284–312 mOsm/kg) and roselle-extract formulations (approx. 280–330 mOsm/kg) [45]. Such values are consistent with the “gold standard” for blood plasma compatibility (~275–295 mOsm/kg), ensuring optimal gastric emptying and intestinal absorption [22]. Notably, our results confirm that using natural fruit juices (10–15% *v*/*v*) allows for maintaining these parameters without the need for synthetic osmotic agents, which is a significant advantage in the context of the “clean label” trend.

A key differentiator of the developed beverages is their high antioxidant potential. In our formulations, the total polyphenol content (TP) reached up to 163 mg GAE/100 mL in the apple–chokeberry (JA) variant. This value is remarkably high compared to the maqui–lemon isotonic blends, which exhibited approximately 81 mg GAE/100 mL [46]. It is well known that chokeberry fruits are a rich source of bioactive such as polyphenols (anthocyanins, procyanidins, phenolic acids, flavonols and flavanols) [47]. Other drinks based on red fruit juices (20 Ż—from cranberry, 9 MCzB—from raspberry and elderberry) were also characterized by a high polyphenol content (higher than 120 mg GAE/100 mL). This is consistent with the findings of Nieć-Leśniak et al. [48], who demonstrated that red fruits juices are characterized by a particularly high content of bioactive compounds, which translates into their high antioxidant potential. We did not determine the content of individual polyphenols; however, an analysis of the literature data on the content of compounds from this group in fruit suggests that such a high total polyphenol content in our isotonic drinks is due to the addition of fruit juices. The pasteurization process used may have enhanced the release of polyphenolic compounds from the fruit matrix [49,50].

The superior radical scavenging activity observed in our study (up to 89% DPPH inhibition) correlates probably with the high levels of anthocyanins found in red fruits. According to Jakobek and Seruga, anthocyanins showed the highest influence on antiradical activity in all fruits. Other phenolics with relatively strong influence on the antiradical activity of fruits are quercetin derivatives and caffeic acid derivatives (chlorogenic acid) in chokeberries and blueberries, quercetin derivatives in blackberries, quercetin derivatives and caffeic acid derivatives (chlorogenic acid) in elderberries, and hydroxycinnamic acids in sour cherries [51]. This is consistent with research on other anthocyanin-rich fruits like maqui, açaí, and blackthorn, which are known to effectively neutralize reactive oxygen species (ROS) generated during intense physical exertion [46]. Furthermore, as suggested by Bendaali et al. [22], the use of organic grape juice and herbal extracts (hops, tea, mint) can further enhance these antioxidant defenses. The strong, positive linear correlation between total phenolic content (TP) and antiradical activity (AA DPPH) suggests that higher concentrations of polyphenols reliably result in greater free radical neutralization.

Our analysis of the Lab* color space revealed a significant negative correlation between lightness (L*) and antioxidant potential (r = −0.46), suggesting that darker, more pigment-dense beverages offer higher health benefits. This phenomenon was also observed in grape juice-based drinks, where water mineralization and pH levels significantly influenced color intensity and anthocyanin stability over time [22].

All samples that were developed and subjected to sensory analysis received positive ratings (M > 6.0) for individual quality attributes and for overall acceptability. This indicates that the organoleptic qualities are well matched and balanced and may meet consumer expectations, whilst maintaining the appropriate osmolality. The aesthetic appeal of isotonic drinks is a crucial factor for consumer choice. In our study, the highest sensory acceptance was recorded for the quince and elderberry (PCzB) blend (M = 8.1), which also displayed high color saturation. Similarly, sensory evaluations of Ajwa date drinks and carbonated roselle beverages showed high ratings (above 5 on a 7-point scale or 8.2 on a 9-point scale), indicating that natural pigments can successfully replace synthetic dyes [44,46].

While traditional sports drinks focus solely on fluid replacement, our results—supported by the literature—suggest that juice-based isotonics may offer therapeutic benefits. The high polyphenol content in fruit juices has been linked to the inhibition of α-glucosidase and lipase enzymes, potentially supporting weight loss programs and glycemic control in diabetic patients [46,47,52]. Fruit polyphenols also have a cardioprotective effect and platelet aggregation and anti-inflammatory properties, as well as the ability to increase the high-density lipoprotein (HDL)/low-density lipoprotein (LDL) cholesterol ratio [53]. By incorporating these functional ingredients, as seen, e.g., in our apple–chokeberry and apple–bird cherry variants, isotonic drinks can transition from simple rehydration tools to sophisticated functional foods that support post-exercise recovery and overall metabolic health.

## 5. Conclusions

Functional beverages have recently emerged as one of the fastest-growing food market categories. They comprise products that, in addition to basic nutritional functions, exhibit health-promoting effects due to the presence of bioactive compounds. Consumers are increasingly seeking natural, less processed products with short and understandable labels (“clean label”), as well as items targeted to specific demands, such as physical activity, body recovery, or mental focus. In our study, new isotonic beverages, based on natural juices, were evaluated for their sensorial attributes and antioxidant properties. We found that combining different fruit types (e.g., Japanese quince with elderberry) optimizes both the sensory profile and the antioxidant activity of the isotonic drinks. High concentrations of polyphenols, especially in drinks based on red fruits’ juices, may provide a protective effect against oxidative stress, distinguishing natural isotonics from synthetic commercial competitors. However, further research on the evaluation of specific polyphenol contents and their in vivo biological activity and bioavailability is necessary to verify their beneficial effects for sports, nutrition, and health.

## Figures and Tables

**Table 1:** Characteristics of the isotonic drinks studied.

Sample Code	The Type of Juices Used in the Preparation	Osmolality
**22 M**	raspberry	277.50
**20 Ż**	cranberry	305.25
**16 GP**	pear, Japanese quince	281.50
**9 MCzB**	raspberry, elderberry	307.50
**17 WM**	cherry, raspberry	303.25
**5 PCzB**	Japanese quince, elderberry	288.25
**19 JŻ**	apple, cranberry	312.00
**16 JCz**	apple, bird cherry	289.75
**10 JP**	apple, Japanese quince	288.25
**13 JA**	apple, chokeberry	284.00

Source: own elaboration.

**Table 2:** Results of sensory analysis of innovative fruit juice-based isotonic drinks.

Sample Code Number/Sample	Sensory Differentiator	Mean ± SD	Me	Mo
**114/22 M**	Color	7.2 ± 2.14	7.5	7
	Fragrance	6.8 ± 1.52	8	10
	Sweet taste	7.1 ± 1.73	7.5	8
	Sour taste	7.1 ± 2.07	8	8
	Salty taste	7.6 ± 2.05	8	10
	Bitter taste	8.1 ± 2.41	10	10
	Fruity taste	7.8 ± 2.15	8.5	9
	Clarity	7.1 ± 0.99	7	7
	Bitterness	7.1 ± 2.32	8	9
	Palatability	7.4 ± 1.71	8	8
	Overall acceptance	7.7 ± 1.70	8	8
**226/20 Ż**	Color	7.2 ± 1.87	7.5	5
	Fragrance	7.6 ± 2.25	8	8
	Sweet taste	7.2 ± 1.55	7	7
	Sour taste	7.9 ± 1.45	8	6
	Salty taste	8.4 ± 1.43	9	9
	Bitter taste	8.4 ± 2.01	10	10
	Fruity taste	7.8 ± 1.48	8.5	9
	Clarity	7.3 ± 1.57	7	9
	Bitterness	7.8 ± 1.23	8	8
	Palatability	7.7 ± 1.83	8	8
	Overall acceptance	7.8 ± 1.55	8	9
**336/16 GP**	Color	8.2 ± 2.15	9	9
	Fragrance	8.3 ± 1.83	8.5	10
	Sweet taste	7.0 ± 1.76	7	7
	Sour taste	7.6 ± 1.51	8	9
	Salty taste	7.8 ± 2.24	8.5	10
	Bitter taste	8.4 ± 2.14	10	10
	Fruity taste	6.9 ± 2.04	7.5	8
	Clarity	8.3 ± 1.57	8.5	10
	Bitterness	7.6 ± 2.11	9	9
	Palatability	7.4 ± 1.07	7	7
	Overall acceptance	7.3 ± 1.06	7.5	8
**443/9 MCzB**	Color	6.9 ± 1.19	7.5	7
	Fragrance	7.6 ± 2.27	7.5	10
	Sweet taste	7.6 ± 2.07	8	8
	Sour taste	7.4 ± 1.14	8	8
	Salty taste	7.8 ± 2.20	8	10
	Bitter taste	8.2 ± 1.97	9.5	10
	Fruity taste	7.2 ± 1.62	8	8
	Clarity	7.0 ± 1.79	7.5	7
	Bitterness	8.3 ± 1.64	8.5	10
	Palatability	7.1 ± 0.99	7	7
	Overall acceptance	7.4 ± 0.97	7.5	8
**559/17 WM**	Color	7.3 ± 1.57	7	7
	Fragrance	7.1 ± 0.99	7	7
	Sweet taste	7.1 ± 1.37	7	6
	Sour taste	7.7 ± 1.06	8	8
	Salty taste	7.0 ± 1.79	7.5	7
	Bitter taste	8.3 ± 2.17	9.5	10
	Fruity taste	7.0 ± 1.49	7	7
	Clarity	7.4 ± 1.26	7	7
	Bitterness	6.6 ± 1.71	7	7
	Palatability	7.1 ± 0.88	7	7
	Overall acceptance	7.4 ± 1.35	8	8
**661/5 PCzB**	Color	9.4 ± 0.70	9.5	10
	Fragrance	7.7 ± 2.06	8	8
	Sweet taste	7.2 ± 2.62	7.7	10
	Sour taste	7.1 ± 1.92	8	8
	Salty taste	7.8 ± 1.10	8.5	10
	Bitter taste	7.9 ± 1.51	9.5	10
	Fruity taste	8.1 ± 2.18	8.5	8
	Clarity	8.7 ± 1.79	10	10
	Bitterness	7.9 ± 1.11	9.5	10
	Palatability	7.3 ± 2.15	8	7
	Overall acceptance	8.1 ± 1.45	8	8
**772/19 JŻ**	Color	7.0 ± 1.83	8	8
	Fragrance	8.3 ± 1.49	9	9
	Sweet taste	7.3 ± 0.95	7	7
	Sour taste	7.3 ± 1.16	7.5	8
	Salty taste	7.1 ± 2.28	8	9
	Bitter taste	8.2 ± 1.74	9	10
	Fruity taste	7.0 ± 2.11	6.5	9
	Clarity	7.2 ± 1.32	7	6
	Bitterness	7.3 ± 1.89	7.5	7
	Palatability	7.0 ± 1.63	8	8
	Overall acceptance	7.9 ± 0.88	8	8
**889/16 JCz**	Color	7.6 ± 1.26	8	8
	Fragrance	7.1 ± 1.79	7	7
	Sweet taste	7.2 ± 2.10	7.5	7
	Sour taste	7.0 ± 1.33	7	6
	Salty taste	6.9 ± 1.88	8	8
	Bitter taste	7.0 ± 1.56	8	10
	Fruity taste	7.0 ± 0.67	7	7
	Clarity	7.9 ± 1.77	9	9
	Bitterness	7.2 ± 2.22	7.5	7
	Palatability	7.4 ± 1.26	7	6
	Overall acceptance	7.8 ± 0.92	8	8
**990/10 JP**	Color	8.2 ± 2.30	9.5	10
	Fragrance	7.8 ± 1.99	8.5	8
	Sweet taste	7.2 ± 2.225	7	4
	Sour taste	7.6 ± 1.96	8.5	9
	Salty taste	7.5 ± 2.12	8	8
	Bitter taste	8.2 ± 1.18	9.5	10
	Fruity taste	7.1 ± 1.45	7.5	8
	Clarity	8.3 ± 1.79	9.5	10
	Bitterness	8.1 ± 2.18	8.5	10
	Palatability	7.7 ± 1.77	8	8
	Overall acceptance	7.8 ± 1.87	9	9
**100/13 JA**	Color	7.3 ± 1.16	8	8
	Fragrance	7.0 ± 1.89	7	7
	Sweet taste	7.2 ± 1.40	7.5	8
	Sour taste	7.0 ± 1.63	7	7
	Salty taste	7.7 ± 2.00	8	7
	Bitter taste	7.4 ± 1.60	9	10
	Fruity taste	7.0 ± 0.94	7	6
	Clarity	7.7 ± 1.21	8	8
	Bitterness	7.0 ± 1.16	7	7
	Palatability	7.0 ± 0.94	7	7
	Overall acceptance	6.9 ± 1.29	7	7

**Table 3:** The average values (±SD) of the individual physicochemical parameters.

Sample Code	TP [mg GAE/100 mL]	AA DPPH [%]	L*	a*	b*
**22 M**	50.39 ± 3.45 a	49.05 ± 2.93 a	28.16 ± 0.10 f	4.95 ± 0.03 a	3.58 ± 0.08 g
**20 Ż**	122.11 ± 6.30 b	87.94 ± 8.19 a	21.36 ± 0.33 e	1.74 ± 0.06 b	0.95 ± 0.05 a
**16 GP**	55.96 ± 2.46 a	56.68 ± 3.93 a	28.88 ± 0.39 a	−0.70 ± 0.06 c	0.28 ± 0.08 f
**9 MCzB**	124.53 ± 18.49 b	80.32 ± 1.72 a	25.03 ± 0.34 c,d	6.16 ± 0.03 d	2.02 ± 0.12 a,d
**17 WM**	57.97 ± 9.48 a	57.08 ± 3.58 a	25.50 ± 0.09 g	−0.12 ± 0.03 e	2.60 ± 0.08 h
**5 PCzB**	65.49 ± 12.20 a	54.98 ± 3.76 a	31.83 ± 0.17 a	7.70 ± 0.02 f	3.06 ± 0.10 b,c
**19 JŻ**	35.16 ± 3.39 a	45.28 ± 2.86 a	24.64 ± 0.33 c	0.29 ± 0.03 g	2.17 ± 0.13 a,e
**16 JCz**	47.86 ± 9.73 a	47.78 ± 5.08 a	27.16 ± 0.03 h	0.97 ± 0.06 h	2.13 ± 0.08 c,d
**10 JP**	104.02 ± 2.57 b	87.62 ± 6.94 a	21.63 ± 0.03 d	2.10 ± 0.05 i	1.64 ± 0.05 e
**13 JA**	163.26 ± 11.30 c	89.74 ± 0.33 a	23.03 ± 0.03 b	1.95 ± 0.03 j	1.30 ± 0.51 b

Source: own work. Different letters (a–j) show significant statistical differences between mean values at *p* ≤ 0.05 error level according to Tukey’s test.

## Data Availability

The original contributions presented in this study are included in the article. Further inquiries can be directed to the corresponding author.
